# The Potential Role of *Azadirachta indica* Treatment on Cisplatin-Induced Hepatotoxicity and Oxidative Stress in Female Rats

**DOI:** 10.1155/2013/741817

**Published:** 2013-12-04

**Authors:** Mohamed A. Dkhil, Saleh Al-Quraishy, Ahmed M. Aref, Mohamed S. Othman, Kamal M. El-Deib, Ahmed E. Abdel Moneim

**Affiliations:** ^1^Department of Zoology, College of Science, King Saud University, Riyadh 11451, Saudi Arabia; ^2^Department of Zoology and Entomology, Faculty of Science, Helwan University, Cairo 11795, Egypt; ^3^Biological Science Department, Faculty of Dentistry, Modern Science and Arts University (MSA), Giza 12111, Egypt; ^4^Biochemistry and Molecular Biology Department, Faculty of Pharmacy, Modern Science and Arts (MSA), Giza 12111, Egypt; ^5^Molecular Drug Evaluation Department, National Organization for Drug Control & Research (NODCAR), Giza 12553, Egypt; ^6^Department of Biochemistry and Molecular Biology, Asturias Institute of Biotechnology, University of Oviedo, Oviedo 33006, Spain

## Abstract

*Azadirachta indica* A. Juss. (neem, family: Meliaceae) is perhaps the most commonly used traditional medicinal plant of India. In this study we investigated the protective effect of methanolic neem leaves extract (MNLE; 500 mg/Kg bwt) on rats treated with cisplatin (CDDP)-induced hepatotoxicity. Adult rats were randomly divided into four groups. CDDP was given to rats by intraperitoneal injection, while MNLE was given by oral gavage for 5 days after the CDDP injection. The injury and oxidative stress caused by CDDP on the liver and the effect of MNLE were evaluated by measuring (a) histological changes, (b) tissue biochemical oxidant and antioxidant parameters, and (c) investigating apoptosis markers immunohistochemically and by real time PCR. After treatment with MNLE, the histological damage and apoptosis induction caused by cisplatin were improved. Malondialdehyde and nitric oxide were significantly decreased; the antioxidant system, namely, glutathione content, glutathione-S-transferase, glutathione peroxidase, catalase, and superoxide dismutase activities were significantly elevated. In conclusion, MNLE may have a potential role when combined with cisplatin in chemotherapy to alleviate cisplatin-induced damage and oxidative stress in liver.

## 1. Introduction

Cisplatin, cis-diamminedichloroplatinum (CDDP), with the molecular formula cis-[Pt(NH_3_)_2_Cl_2_], is one of the most remarkable platinum-based drug used in “the war on cancer” [1–3]. CDDP and related platinum-based therapeutics are being used for the treatment of testicular, head and neck, ovarian, cervical, nonsmall cell lung carcinoma, and many other types of cancer. Its use is mainly limited by two factors: acquired resistance to CDDP and severe side effects in normal tissues [4].

The side effects induced by CDDP include neurotoxicity, ototoxicity, nausea and vomiting, and nephrotoxicity. During the aggressive treatment protocols, higher doses of CDDP that may be required for effective tumor suppression could also lead to hepatotoxicity, which is also encountered during low-dose repeated CDDP therapy [5].

The liver is highly susceptible to oxidative reactions as it is the main center of metabolism of most of the substances in the body, including exogenous substances like drugs. Usually nephrotoxicity is monitored during treatment with CDDP, but hepatotoxicity does not receive much attention [6]. It has been reported that oxidative stress through the generation of reactive oxygen species (ROS) decreased antioxidant defense systems, including antioxidant enzymes and nonenzymatic molecule glutathione (GSH), which are all major aspects of CDDP toxicity [7, 8].

In addition, functional and structural mitochondrial damage, apoptosis, perturbation in Ca^2+^ homeostasis, and involvement of proinflammatory genes such as COX-2 and inducible nitric oxide synthase (iNOS) may play some important roles in the mechanism of CDDP hepatotoxicity [9].

Neem tree (*Azadirachta indica*) is one of the most widely used medicinal plants in the world [10] and has been for many years. The importance of the neem tree has been recognized by the US National Academy of Sciences. It published a report in 1992 entitled, “Neem—a tree for solving global problem” [11]. Biswas et al. [12] have reviewed the biological activities of some of the neem compounds, pharmacological actions of the neem extracts, clinical study, and plausible medicinal applications of neem along with their safety evaluation.

The leaves of the neem tree are traditionally used as medicinal preparations for their immunomodulatory, anti-inflammatory, antihyperglycemic, antiulcer, antimalarial, antifungal, antibacterial, antiviral, antimutagenic, and anticarcinogenic properties [13]. We aimed in this work to evaluate the hepatoprotective efficacy of a natural product, neem, against CDDP-induced hepatotoxicity.

## 2. Materials and Methods

### 2.1. Chemicals

Cisplatin was purchased from Sigma (St. Louis, MO, USA). Perchloric acid, thiobarbituric acid (TBA), and trichloroacetic acid (TCA) were purchased from Merck. All other chemicals and reagents used in this study were of analytical grade. Double-distilled water was used as the solvent.

### 2.2. Preparation of Neem Leaves Extract

Fresh matured leaves of neem tree were collected from a garden in Obour City, Cairo, on August 2011. The samples were identified in Botany Department, Faculty of Science, Helwan University. The leaves were cleaned, dried, and powdered. Powder (100 g) of *A. indica* leaves was then consecutively macerated for one day in petroleum ether, ethyl acetate, chloroform, and methanol, respectively. On the basis of the preliminary phytochemicals tests conducted, the methanol extract was found to be rich in terms of chemical constituents, and therefore was selected for the experiment. The methanol was removed under reduced pressure to obtain a semisolid mass of methanolic neem leaves extract (MNLE). The MNLE was then stored in −20°C until used.

### 2.3. Animals and Experimental Design

Adult females of Wister albino rats weighing 150–180 g were obtained from The Holding Company for Biological Products and Vaccines (VACSERA, Cairo, Egypt). Rats were provided with water and balanced diet *ad libitum*. The experiments were approved by the state authorities following the Egyptian rules on animal protection. Twenty-four adult rats were randomly divided into four groups, six rats per group. Group I (Con group) served as untreated control. Group II (CDDP group) received a single intraperitoneal injection of CDDP (5 mg/kg, Sigma) and left for 5 days. Group III (MNLE group) received an oral administration of 500 mg/kg MNLE for 5 days *via *epigastric tube. Group IV (CDDP-N group) received the same dose of the extract for 5 days after a single intraperitoneal injection of CDDP (5 mg/kg). The animals of all groups were sacrificed by fast decapitation; blood samples were collected, allowed to stand for half an hour, and then centrifuged at 500 g for 15 min at 4°C to separate serum and stored at −70°C for the different biochemical measurements. The liver was dissected out. Part of the liver tissue was fixed immediately in 10% phosphate buffered formaldehyde for histological and immunohistochemical studies. Another part was weighed and homogenized immediately to give 50% (w/v) homogenate in ice-cold medium containing 50 mM Tris-HCl, and pH 7.4, then centrifuged at 500 g for 10 min at 4°C. The supernatant was used for the various biochemical determinations.

### 2.4. Liver Function Tests

Colorimetric determination of alanine aminotransferase (ALT) or aspartate aminotransferase (AST) was estimated by measuring the amount of pyruvate or oxaloacetate produced by forming 2,4-dinitrophenylhydrazine according to the method of Reitman and Frankel [14]. The color of which was measured spectrophotometerically at 546 nm. *γ*-Glutamyl transpeptidase (*γ*GT) and alkaline phosphatase were assayed in liver homogenate using kits provided by Biodiagnostic Co. (Giza, Egypt), according to the method described by Szasz [15] and Belfield and Goldberg [16], respectively. Also, total bilirubin (TB) in serum was assayed according to the method of King and Coxon [17].

### 2.5. Oxidative Stress Markers

Homogenates of liver were used to determine lipid peroxidation (LPO) by reaction of thiobarbituric acid [18]. Similarly, those homogenates were used to determine nitrite/nitrate (nitric oxide; NO) [19] and glutathione (GSH) [20].

### 2.6. Enzymatic Antioxidant Status

The same homogenates of liver were used in determination of superoxide dismutase (SOD) [21], catalase (CAT) [22], glutathione peroxidase (GPx) [23], and glutathione reductase (GR) [24] activities.

### 2.7. Real Time PCR

Total RNA was isolated from the liver tissue using an RNeasy Plus Minikit (Qiagen, Valencia, CA). One microgram total RNA and random primers were used for cDNA synthesis using the RevertAid H Minus Reverse Transcriptase (Fermentas, Thermo Fisher Scientific Inc., Canada). For real time PCR analysis, the cDNA samples are run in triplicate and *β*-actin is used as reference gene. Each PCR amplification includes nontemplate controls containing all reagents except cDNA. Real time PCR reactions were performed using Power SYBR Green (Life Technologies, CA) and was conducted on the Applied Biosystems 7500 Instrument. The typical thermal profile is 95°C for 3 min, followed by 40 cycles of 95°C for 15 s and 56°C for 30 s. After PCR amplification, the ΔCt is calculated by subtraction of the *β*-actin Ct from each sample Ct. The method of Pfaffl was used for data analysis. The PCR primers for Bax and caspase-3 and 9 genes were synthesized by Jena Bioscience GmbH (Jena, Germany). Primers were designed using Primer-Blast program from NCBI. The PCR primer sequences are BLAST, searched to ensure for specificity to this particular gene. For a reference gene, the *β*-actin is used. The primer sets used were rat caspase-3 (forward: GCATGATCCGCGACGTGGAA; reverse: AGATCCATGCCGTTGGCCAG), caspase-9 (forward: ATGCAGGTCCCTGTCATG; reverse: GCTTGAGGTGGTTGTGGA), Bax (forward: AGATCACATTCACGGTGCTG; reverse: CTTCAGAGGCAGGAAACAGG), and *β*-actin (forward primer: AGAGGGAAATCGTGCGTGAC; reverse: CAATAGT GATGACCTGGCCGT).

### 2.8. Histopathological Examination

Tissue samples were fixed in 10% neutral formalin for 24 hours, and paraffin blocks were obtained and routinely processed for light microscopy. Slices of 4-5 *μ*m were obtained from the prepared blocks and stained with hematoxylin-eosin. The preparations obtained were visualized using a Nikon microscopy at a magnification of 400x.

### 2.9. Immunohistochemistry for Detection of NF-*κ*B

For immunohistochemistry, liver sections (4 *μ*m) were deparaffinized and then boiled to unmask antigen sites; the endogenous activity of peroxidase was quenched with 0.03% H_2_O_2_ in absolute methanol. Liver sections were incubated overnight at 4°C with a 1 : 200 dilution of mouse NF-*κ*B antibodies (Santa Cruz CA, USA) in phosphate buffered saline (PBS). After removal of the unbound primary antibodies by rinsing with PBS, slides were incubated with a 1 : 500 dilution of biotinylated anti-mouse secondary antibody. Bound antibodies were detected with avidin biotinylated peroxidase complex ABC-kit Vectastain, and the chromogen 3,3′-diaminobenzidine tetrachloride (DAB) is used as substrate. After appropriate washing in PBS, slides were counterstained with hematoxylin. All sections were incubated under the same conditions with the same concentration of antibodies and at the same time; so the immunostaining was comparable among the different experimental groups.

### 2.10. Statistical Analysis

Differences between obtained values (means ± SEM, *n* = 6) were carried out by one way analysis of variance (ANOVA) followed by the Duncan test. A *P* value of 0.05 or less was taken as a criterion for a statistically significant difference.

## 3. Results

**Figure 1:**
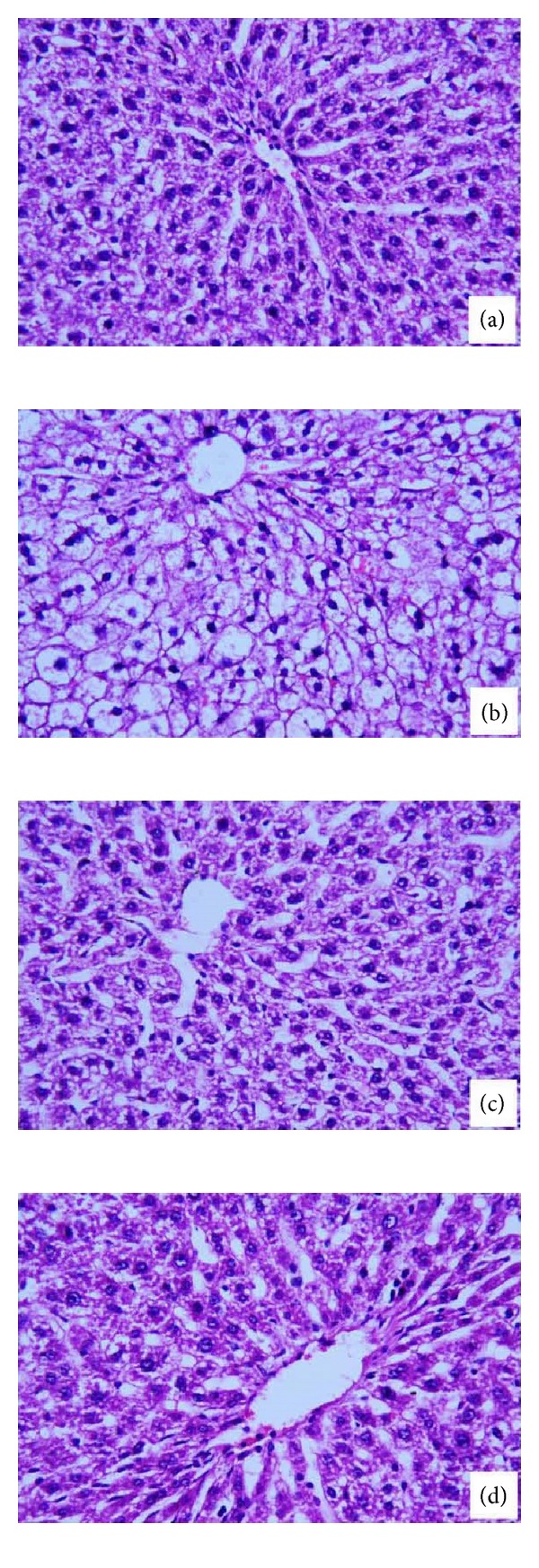
Histological changes in the liver of rats. (a) A control liver with normal architecture. (b) Rats treated with cisplatin with prominent inflammation and hepatocytic vacuolation. (c) Rats treated with the neem leaves extract for 5 days. (d) Rats treated with the cisplatin and neem leaves extract. Sections were stained with hematoxylin and eosin (400x).

Normal control animals have revealed clear cut hepatic lobules separated by interlobular septa, transversed by portal vein (Figure 1(a)). The CDDP-induced hepatic damage is characterized by dispersed areas of necrotic hepatocytes, inflammatory cellular infiltration cytoplasmic vacuolation, and degeneration of hepatocytes (Figure 1(b)). Treatment of rats with MNLE largely prevented CDDP-induced histopathological changes in the liver as indicated by a reduction in inflammatory cellular infiltration and hepatocytic damages (Figure 1(d)). These histological abnormalities is coincide with a significant increase in activity of ALT, AST, *γ*GT, ALP, and TB levels (Table 1), while treatment with MNLE significantly restored these levels to normal values (*P* < 0.05).

**Figure 2:**
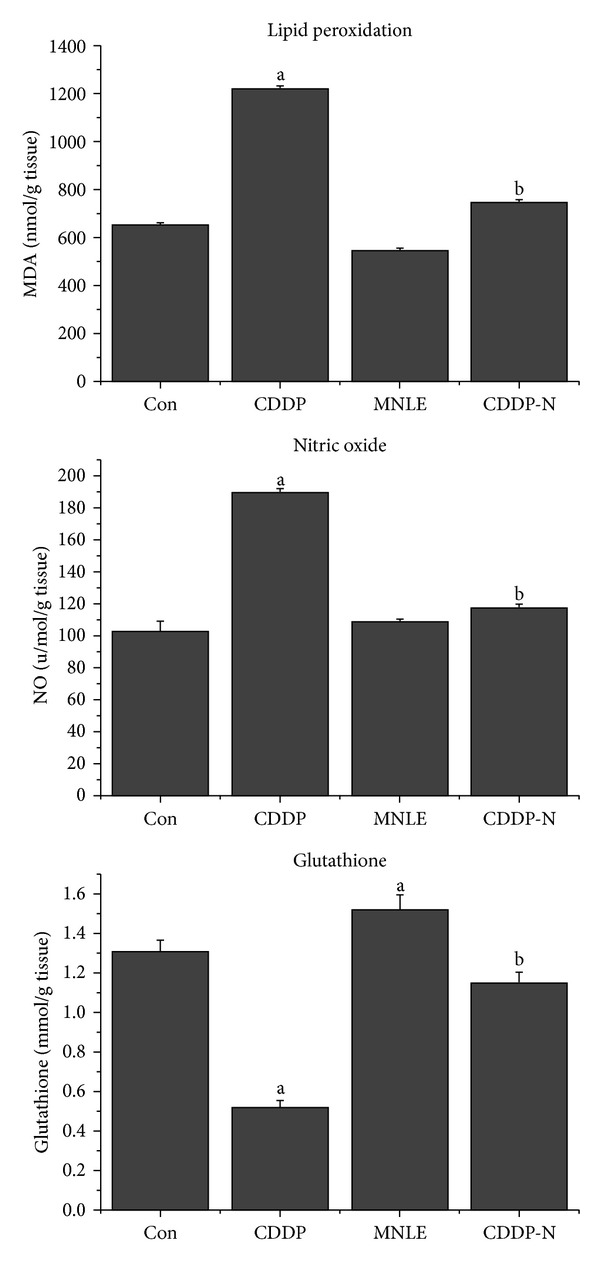
Protective effects of neem leaves extract on cisplatin-induced elevation in lipid peroxidation, nitric oxide levels, and reduction in glutathione level in liver of rats. Values are means ± SEM (*n* = 6). ^a^ *P* < 0.05, significant change with respect to **Control**; ^b^ *P* < 0.05, significant change with respect to **CDDP **group.

The CDDP-induced hepatic oxidant stress was evident by increased lipid peroxidation and nitric oxide and decreased GSH content as shown in (Figure 2). The LPO and NO levels in the liver of animals that administered CDDP alone were observed to display an increase compared with control group, and this increase was found to be statistically significant. The production of these markers is used as a biomarker to measure the level of oxidative stress in an organism [25]. This increase was attenuated by treatment with MNLE.

**Figure 3:**
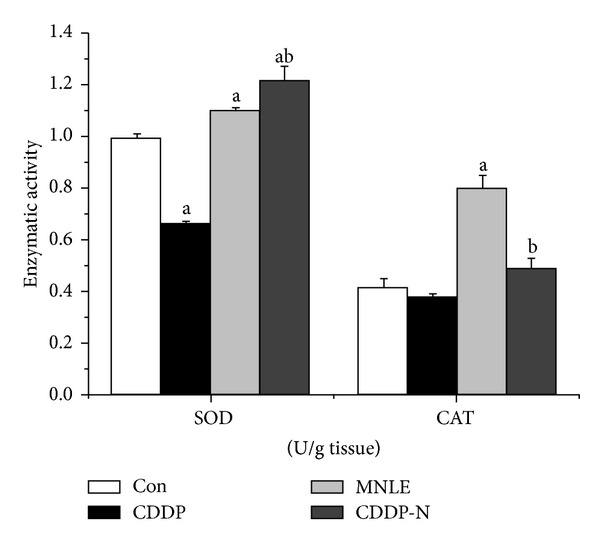
Protective effects of neem leaves extract on cisplatin-induced reduction in superoxide dismutase and catalase activities in liver of rats. Values are means ± SEM (*n* = 6). ^a^ *P* < 0.05, significant change with respect to **Control**; ^b^ *P* < 0.05, significant change with respect to **CDDP **group.

Also significantly reduced activities of the antioxidant enzymes GPx, GST, GR, CAT, and SOD were seen in the liver tissues of CDDP-treated rats compared with the control group. Treatment of rats with MNLE significantly alleviated these CDDP-induced decreases (Table 2, Figure 3) with a significant increase in GSH levels.

**Figure 4:**
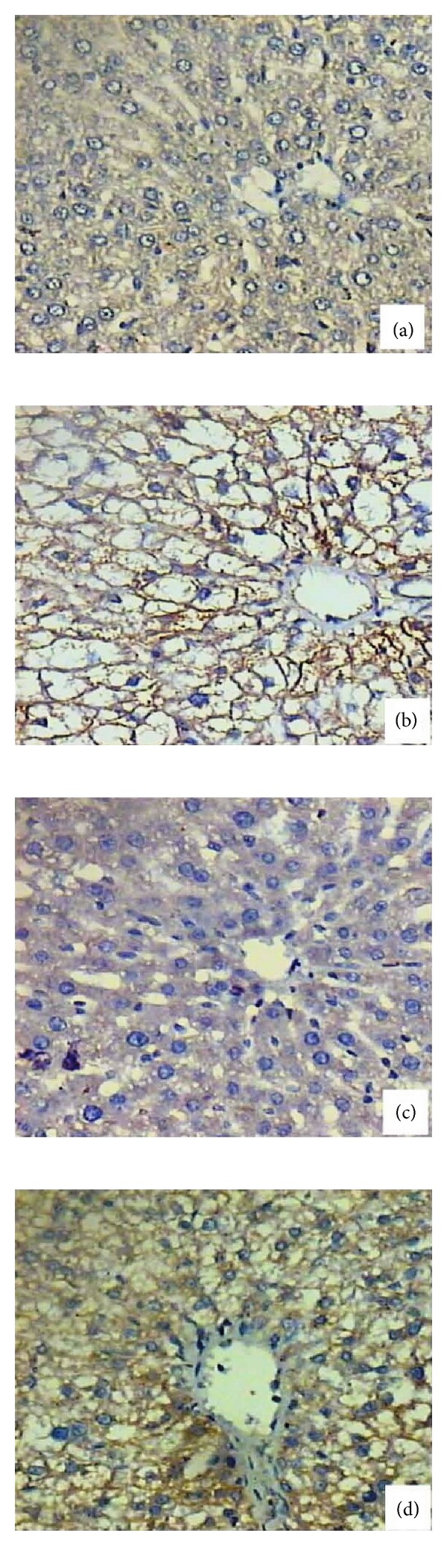
NF-*κ*B expression changes in the liver of rats. (a) Normal liver showing very weak expression for NF-*κ*B. (b) Treated liver with cisplatin showing positive expression for NF-*κ*B. (c) Neem treated liver showing negative expression for NF-*κ*B. (d) Treated liver with cisplatin and MNLE showing medium expression for NF-*κ*B (400x).

NF-*κ*B is a redox-sensitive transcription factor that has been proposed to be the sensor for oxidative stress [26]. The immunostaining activity for NF-*κ*B was increased in CDDP group compared with control indicating the oxidative stress effect induced by CDDP, while administration of MNLE decreased the number of immunostained cells indicating its antioxidant effect (Figure 4).

**Figure 5:**
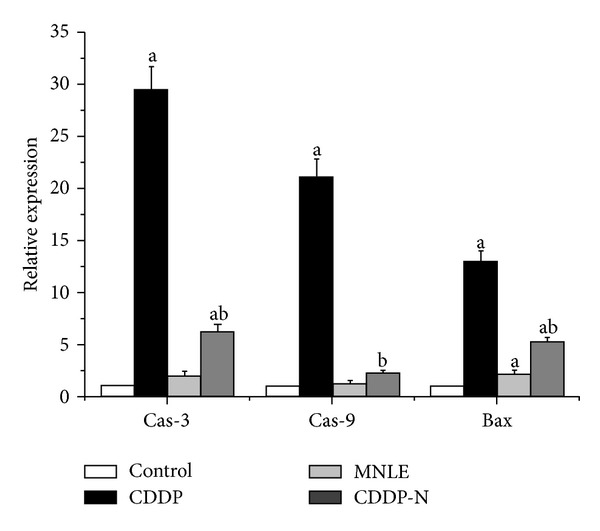
Relative quantification using RT-qPCR of mRNA expression of caspase-3, 9, and Bax genes in liver of rats treated with cisplatin and neem leaves extract. ^a^ *P* < 0.05, significant change with respect to **Control**; ^b^ *P* < 0.05, significant change with respect to **CDDP** group.

In this study, apoptosis in the liver was investigated with PI; comparison of apoptotic activities groups are shown in supplementary data, (see Figure 1 in Supplementary Material available online at http://dx.doi.org/10.1155/2013/741817); we observed that the number of apoptotic cells were increased in the liver of CDDP group when compared with other groups, but in MNLE and CDDP group, there were highly decreased in apoptotic cell numbers, which was confirmed by the results of real time PCR that have shown that there are increases in the expression of the proapoptotic gene Bax and the caspases-3 and 9 in the liver treated with CDDP, while treatment with MNLE has decreased the expression of these genes (Figure 5).

## 4. Discussion

Different strategies have been proposed to inhibit CDDP-induced toxicity [27]. Development of therapies to prevent the action of generation of free radicals may influence the progression of oxidative liver damage induced by CDDP. Recent studies suggest that using plant derived chemopreventive agents in combination with chemotherapy can enhance the efficacy of chemotherapeutic agents and lower their toxicity to normal tissues [28, 29].

Neem is one of those candidate plants which has chemoprotective effect and strong antioxidant potential [30, 31]. The components of the neem tree, like bark, seed, leaf, fruit, gum, oil, and so forth, contain compounds offering some impressive therapeutic applications [12]. There are several reported active compounds in neem plant, like nimbin, azadirachtin, nimbidiol, quercetin, and nimbidin [32, 33]. In a study by Mallick et al. [34] they confirmed nontoxic effect of neem leaves extract on rat liver and kidney, even in higher doses exceeding the effective dose.

The present study has evaluated the effect of MNLE on rats with CDDP-induced hepatotoxicity. Earlier experimental studies have shown that a minimum dose of CDDP (5 mg/kg bwt, i.p) was sufficient to induce hepato and nephrotoxicity in rats [35–37].

The liver is known to accumulate significant amounts of CDDP, second only to the kidney [38]; thus hepatotoxicity can be associated with CDDP treatment [35]. Our study showed many histopathological and biochemical abnormalities in the liver of CDDP-injected animals with single dose (5 mg/kg bwt), which is inconsistent with the previous results [38, 39]. Moreover, the treatment with MNLE (500 mg/kg) for 5 days ameliorated CDDP-induced liver damages associated with free radical production [5, 6] by enhancing the enzyme activity to normal values and preserving the liver parenchyma; that is, the appearance of the hepatocytes, sinusoids, Von Kupffer cells, and the portal triad was similar to the control rats; these results are inconsistent with the previous studies [40, 41].

El-Sayyad et al. [38] reported that light microscopic observations revealed that CDDP caused hepatotoxicity, including dissolution of hepatic cords, focal inflammation and necrotic tissues, periportal fibrosis, degeneration of hepatic cords, and increased apoptosis. It is well known that CDDP induces oxidative stress [42]. Indeed, some recent studies have suggested that oxidative stress plays an important mechanism in CDDP-induced hepatotoxicity [5, 43–46].

Reactive oxygen species such as hydrogen peroxide, superoxide anions, and hydroxyl radicals are generated under normal cellular conditions and are immediately detoxified by major scavenger enzymatic and nonenzymatic molecules [47]. However, excessive ROS production by CDDP causes antioxidant imbalance and leads to lipid peroxidation and antioxidant depletion [48].

In our study, the major scavenger enzymes activities (SOD, CAT, GPx, GST, and GR) were significantly decreased in liver of CDDP -treated rats our results are in agreement with results obtained earlier [39, 49, 50]. This finding can be explained by CDDP-induced increase in free radical generation or a decrease in amounts of protecting enzymes against lipid peroxidation [49].

Cisplatin is accumulated in its target organs by covalently binding with their proteins [51]. This can affect their antioxidant enzymes which are the first line of defense against any oxidative insult to the cells. CDDP caused nephrotoxicity and hepatotoxicity evidenced by marked decline in activity of the antioxidant enzymes [52].

CDDP induces nitrosative stress by NO production as secondary event following increase in inducible nitric oxide synthase (iNOS) [53]. In the current study NO production in the CDDP-treated group was significantly higher than that in the control group; our results are consistent with the results obtained by Kart et al. [39], who have shown that there is strong immunoreactivity against iNOS in the liver tissue of the CDDP-treated group. Chirino et al. [54] reported that inhibition of iNOS reduced the CDDP-induced renal damage and nitrosative stress.

Exogenous and endogenous protective agents with antioxidant properties were reported to show some protective effects in CDDP-induced hepatotoxicity. Neem is one promising agent against various toxicities associated with oxidative stress and peroxidative damage. Neem was shown to have prominent antioxidant, radical-scavenging, and antiperoxidative activities [55]. In the current study, MNLE (500 mg/kg) for 5 days of treatment ameliorated CDDP toxicity, indicated by significant reduction in the elevated LPO and NO levels and also normalized tissue GSH level.

In the present study, injection of CDDP to female rats resulted in elevating functional markers of liver in the serum. This is a clear indication of hepatotoxicity caused by CDDP as the markers are released by the damaged organs in the circulatory system. A marked recovery was observed in the markers of liver function after combination treatment by CDDP with MNLE. Several possible mechanisms have been proposed to explain the pathological status of liver after CDDP treatment [56, 57].

Identification of agents that inhibit carcinogen activation, phase II detoxification, and abrogation of NF-*κ*B signaling has become a major focus of cancer chemoprevention in recent years [58]. Our results revealed that the immunohistochemical expression of NF-*κ*B has shown a strong immunoreactivity in the CDDP-treated group, while treatment with MNLE has decreased the number of positive cells. Our results are consistent with the results obtained by Manikandan et al. [59], who have shown that neem leaf subfraction has a potential role in inhibiting NF-*κ*B.

A mechanism of CDDP toxicity is that CDDP-induces liver cell apoptosis by cytochrome c release and caspase-3 activation [56]. Our results showed that treatment with MNLE has decreased the expression of Bax, caspase-3 and 9.

In conclusion, results of the current study suggest that oxidative stress and lipid peroxidation along with nitrosative stress are important features in CDDP hepatotoxicity. CDDP hepatotoxicity is associated with ROS production and might contribute to oxidative stress. However, treatment with MNLE was found to reduce the CDDP-induced liver chemical changes and apoptotic cell numbers. This may be due to antiapoptotic and antioxidant effects of neem extract [60]. Therefore, we suggest (or propose) that usage of neem leaves extract in combination with CDDP in anticancer therapy may help to reduce the CDDP-induced toxicities.

## Supplementary Material

Morphological changes visualized under fluorescence microscope with PI staining in liver sections of rats treated with cisplatin and neem leaves extract (400×).

## Figures and Tables

**Table 1:** Protective effects of methanolic neem leaves extract on cisplatin (CDDP) induced alternation in liver function of rats.

Groups	ALT (IU/L)	AST (IU/L)	*γ*GT activity (IU/L)	ALP (IU/L)	Total bilirubin (mg/dL)
Con	85.23 ± 3.98	68.98 ± 1.90	31.66 ± 1.15	4.26 ± 0.38	2.78 ± 0.16
CDDP	121.82 ± 3.13^a^	90.32 ± 1.19^a^	54.22 ± 1.88^a^	6.82 ± 0.30^a^	5.87 ± 0.20^a^
MNLE	83.59 ± 1.23	72.09 ± 2.90	29.60 ± 1.24	3.84 ± 0.28	2.57 ± 0.08
CDDP-N	86.28 ± 1.91^b^	70.88 ± 1.67^b^	36.33 ± 1.37^b^	4.99 ± 0.25^b^	3.13 ± 0.22^b^

Values are means ± SEM (*n* = 6).

^a^ *P* < 0.05, significant change with respect to **Control**; ^b^ *P* < 0.05, significant change with respect to **CDDP** for Duncan's post hoc test.

**Table 2:** Protective effects of methanolic neem leaves extract on cisplatin (CDDP) induced alternation in enzymatic antioxidant molecules of rats.

Groups	GR (*μ*mol/hr/g tissue)	GST (*μ*mol/hr/g tissue)	GPx (U/g tissue)
Con	93.77 ± 8.47	0.029 ± 0.006	567.39 ± 34.86
CDDP	73.11 ± 3.18^a^	0.018 ± 0.003^a^	313.41 ± 28.39^a^
MNLE	184.20 ± 7.44^a^	0.030 ± 0.001	459.61 ± 30.88^a^
CDDPN	138.66 ± 4.73^ab^	0.049 ± 0.004^ab^	1350.93 ± 60.83^ab^

Values are means ± SEM (*n* = 6).

^a^ *P* < 0.05, significant change with respect to **Control**; ^b^ *P* < 0.05, significant change with respect to **CDDP** for Duncan's post hoc test.
