# Supplementary material for: The Potential Role of Azadirachta indica Treatment on Cisplatin-Induced Hepatotoxicity and Oxidative Stress in Female Rats
Source: Oxid Med Cell Longev. 2013 Dec 4;2013:741817. doi: 10.1155/2013/741817 (PMC3867870; doi:10.1155/2013/741817)
Supplement: Supplementary file 1 — Morphological changes visualized under fluorescence microscope with PI staining in liver sections of rats treated with cisplatin and neem leaves extract (400×). [file 741817.f1.pdf]

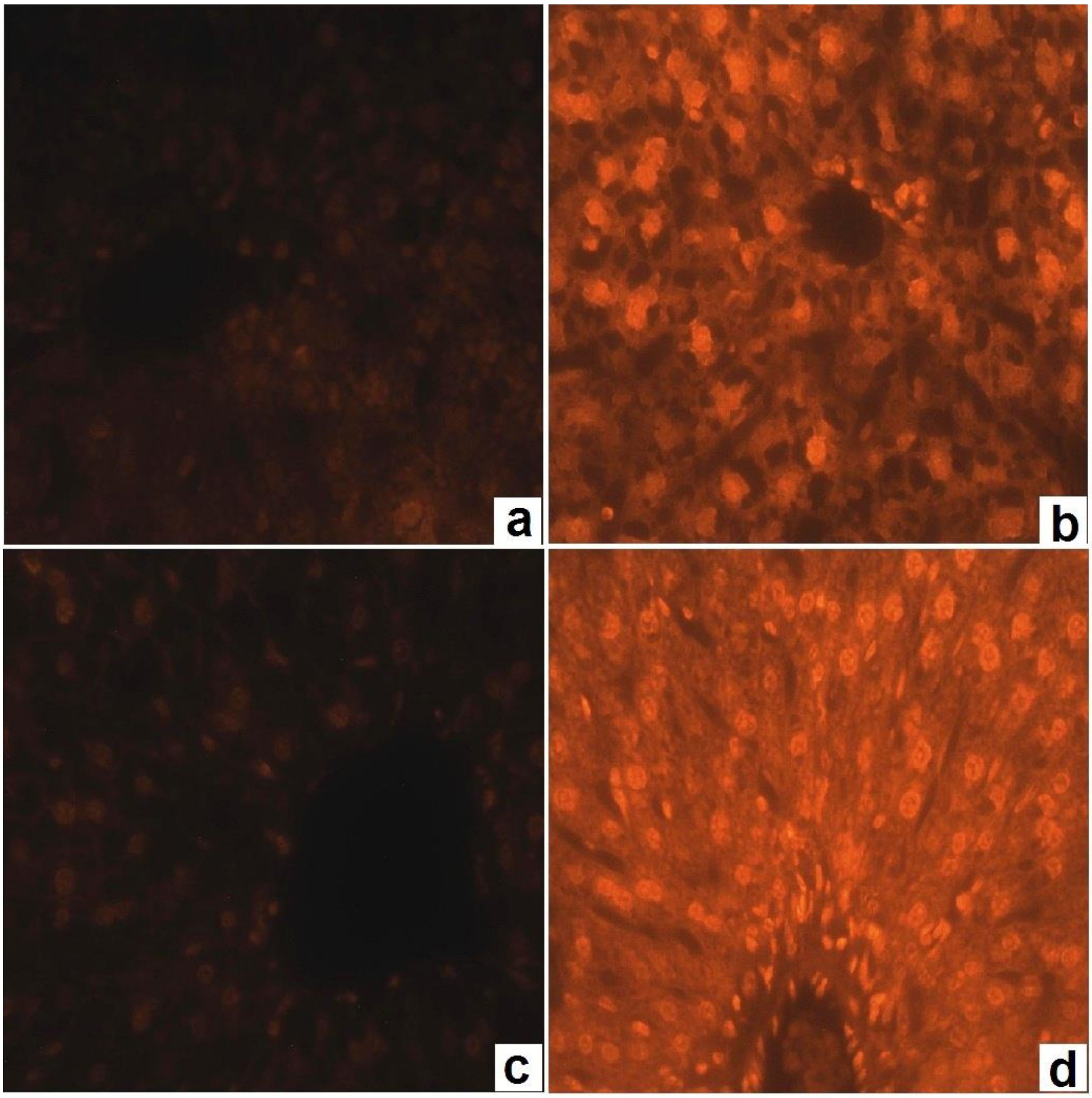

Figure (1): Morphological changes visualized under fluorescence microscope with PI staining in liver sections of rats treated with cisplatin and neem leaves extract (400×).
